# Alcohol use as a risk factor for tuberculosis – a systematic review

**DOI:** 10.1186/1471-2458-8-289

**Published:** 2008-08-14

**Authors:** Knut Lönnroth, Brian G Williams, Stephanie Stadlin, Ernesto Jaramillo, Christopher Dye

**Affiliations:** 1 Stop TB Department, World Health Organization, Geneva, Switzerland

## Abstract

**Background:**

It has long been evident that there is an association between alcohol use and risk of tuberculosis. It has not been established to what extent this association is confounded by social and other factors related to alcohol use. Nor has the strength of the association been established. The objective of this study was to systematically review the available evidence on the association between alcohol use and the risk of tuberculosis.

**Methods:**

Based on a systematic literature review, we identified 3 cohort and 18 case control studies. These were further categorized according to definition of exposure, type of tuberculosis used as study outcome, and confounders controlled for. Pooled effect sizes were obtained for each sub-category of studies.

**Results:**

The pooled relative risk across all studies that used an exposure cut-off level set at 40 g alcohol per day or above, or defined exposure as a clinical diagnosis of an alcohol use disorder, was 3.50 (95% CI: 2.01–5.93). After exclusion of small studies, because of suspected publication bias, the pooled relative risk was 2.94 (95% CI: 1.89–4.59). Subgroup analyses of studies that had controlled for various sets of confounders did not give significantly different results and did not explain the significant heterogeneity that was found across the studies.

**Conclusion:**

The risk of active tuberculosis is substantially elevated in people who drink more than 40 g alcohol per day, and/or have an alcohol use disorder. This may be due to both increased risk of infection related to specific social mixing patterns associated with alcohol use, as well as influence on the immune system of alcohol itself and of alcohol related conditions.

## Background

It has been evident for decades that there is a strong association between alcohol use and risk of tuberculosis (TB). Prevalence of alcohol use disorders among TB patients have ranged from 10% to 50% in studies carried out in Australia, Canada, Russia, Switzerland, and the USA [1-7]. Similar evidence of a strong link emerges from studies in which population groups with high prevalence of alcohol use disorders have been screened for TB. Jones et al[8] found that the prevalence of active pulmonary TB among social service clients (among whom alcohol use disorders was the main problem) in the USA in the 1950s was 55 times the prevalence of the general population (2,220/100,000 vs. 40/100,000). Friedman et al[9] reported a 46 times higher prevalence among people with alcohol use disorders (who did not abuse other drugs) in New York in the early 1980s (1,500/100,000 vs. 32/100,000). In a cohort of persons with alcohol use disorders who were followed prospectively for 8 years, the TB incidence was 464/100,000 person-years, which was 9 times the age-matched incidence among the general population in New York[10]. However, these studies did not control for potential confounders.

Possible causal pathways include specific social mixing patterns among people with alcohol use disorders, leading to higher risk of infection [11-13], or weakened immune system leading to higher risk of break down from infection to TB disease. The latter may be through direct toxic effects of alcohol on the immune system [14-18], or indirectly through micro- and macronutrient deficiency[19], or other alcohol-related medical conditions such as malignancies[20] and depression[21,22].

This paper reviews analytical epidemiological studies with individual-level data on alcohol exposure and TB disease status, with the aim to determine if there is a likely causal association between alcohol use and risk of TB disease. The paper also attempts to estimate the strength of such an association.

## Methods

### Inclusion criteria

The review included case-control and cohort studies that reported individual level data on alcohol exposure (amount of alcohol intake or a clinical diagnosis of an alcohol use disorder) and active TB disease, and which reported either crude or adjusted odds ratio, or crude data from which odds ratios could be calculated.

### Search strategy

Initially, all 16,527 articles in a comprehensive private collection of scientific tuberculosis publications (compiled by Dr Hans Rieder) of which a copy is kept at the Stop TB Department at the World Health Organization, were screened using Reference Manager™, with the keywords "alcohol" or "alcoholism". Next, PubMed was searched using the keywords "alcohol OR alcoholism AND tuberculosis", which revealed a total of 2,007 abstracts. Titles were initially screened, followed by screening of abstracts. In addition, we screened a report of a systematic review of the association between smoking and tuberculosis[23], which included detailed information about all covariates that were analysed in 50 reviewed studies. All studies in which alcohol was a listed covariate were reviewed in detail. Finally, the reference list of all reviewed articles were screened.

### Study assessments and analysis

A total of 21 studies [24-45] fulfilled the inclusion criteria and were further assessed with regards to setting, inclusion criteria of study subjects, definition of exposure and outcome, mechanisms for ascertainment of exposure and outcome, and confounders controlled for (table 1).

**Table 1:** Summary of study characteristics

***Cohort studies***													
**First Author, Year, setting**		**Cohort**		**Outcome measure**		**Exposure Measure**		**Confounders Controlled for**		**Effect size (95% confidence interval)**		**Comments**

Hemilä et al, 1999, Finland, 198–1993		26,975 male smokers participating in RCT on the effect of nutritional support with a-tocopherol, P-carotene, or a-tocopherol + P-carotene for cancer prevention		Clinical diagnoses of TB ascertained from the discharge register of hospitals. 167 incident cases of TB registered from 1985 to 1993.		Self reported at baseline. Alcohol use categorized as 30 gram alcohol per day or more.		Age, BMI, martial status, education, residential neighbourhood, smoking, nutritional intervention		Adjusted relative risk: 1.03 (95% CI: 0.70–1.53)		Eight years follow up and change in drinking pattern not ascertained. Prevalence of exposure among controls: 20%
Moran-Mendoza, 2004, British Columbia, Canada, 1990–2000		33,146 contacts of active TB cases recorded in division of disease control 1990–2000, who had a TST performed, excluding those with TB history and those with HIV, followed until 2001		Any type of TB, registered in the division of TB control database. 228 active cases identified.		Alcoholism as noted in medical record		Age, sex, Canadian-born, aboriginal, DM, malnutrition, malignancy, immunosuppressant treatment, BCG, no of contacts, type of contact, TST size, SES (geographical location), latent TB treatment, intravenous drug use, recent arrival from high TB incidence country		Adjusted relative risk: 2.9 (1.3–6.5)		Entire study population are TB infected. RR reflect risk of progress to active disease. Prevalence of alcoholism among whole cohort: 0.8%
Thomas et al 2005, Tiruvallur district, Tamil Nadu, India, 2000–2001		503 cured new smear positive pulmonary patients as per TB district register, followed prospectively		TB recurrence within 18 months (62 recurrencess recorded)		Self reported during initial treatment. Exposure was "Habitual drinking", which was not defined in terms of amounts or frequency		Adjusted OR from multivariate analysis not reported. Factors accounted for were sex, age, occupation, education, smoking, adherence, drug sensitivity, smear conversion, initial weight		Crude relative risk: 2.3 (1.3–4.1)		Level of exposure not provided, but since the prevalence of exposure of "habitual drinking" in the cohort was 33% in this rural Indian district, it not likely to correspond to high level consumption.

***Case control studies***													

**Author (Year), Setting**		**Cases and controls**		**Exposure Measure**		**Confounders Controlled for**		**Effect size (95% confidence interval)**		**Comments**	

Brown and Campbell. 1961, Hospital for ex-servicemen, Victoria, Australia, 1950s		Cases (100): All consecutive new admissions Controls (100): Randomly selected from surgical ward in same hospital (excluding orthopedic cases)		Self reported daily consumption Moderate to heavy drinking defined as 26 ml alcohol per day or more. Crude numbers for different level of exposure were reported, allowing calculation of association also at the > 50 ml (40 g) and other cut-off points.		Stratified by smoking status. All subjects were men. All ex-army staff in the age bracket 20–70. Age distribution very similar between cases and control. Pre- HIV era		Crude OR of moderate to heavy alcohol vs. none/low: 4.88 (95% CI: 2.59–9.24) For > 50 ml vs =< 50 ml: OR 8.18 (4.05–16.53) For 1–50 ml vs. none: 1.98 (0.89–4.43) Significant (p < 0.0001) dose response relationship: OR 0 (reference) 1.00 10–25 ml/day: 1.66 26–50 ml/day: 2.38 51–75 ml/day: 9.27 76–100 ml/day: 8.50 101–125 ml/day: 27.82 126- ml/day: 43.27		OR not analysed in original study. The ORs reported here are calculated based on crude data reported in the paper Smoking possibly effect modifier. Stratified for none smokers and smokers respectively (any alcohol vs. no alcohol): Non smokers: 2.25 (0.54–9.86) Smokers: 5.22 (1.83–15.61) Prevalence of "moderate to heavy alcohol intake" in controls: 39%	
Lewis and Chamberlain, 1963, Hospital, London, 1962		Cases (100): Male, active cases of pulmonary TB Controls (200): Matched for age and social class: A (100): From medical and surgical wards at the same hospital. B (100): From emergency department at another, general, hospital		Self-reported average daily consumption 6 months before symptoms started "Regular drinking" defined as the equivalent of 2 or more pints per day.		Only men, stratified by age, social class, marital status and smoking. Pre-HIV era.		Crude OR for regular drinkers vs. not regular drinker 2.64 (95% CI: 1.50-4-66) Did not change when stratified for smoking status: OR 2.68 and 2.61 in respective stratum		OR not analysed in original study. Social class effect modifier? Stratified for SES: Class I-II: OR = 1.16 (0.42–3.22) Class III-V: OR = 4.07 (1.98–8.41) Prevalence of "regular drinkers" among controls: 19.5% UK pint = 568 ml. 2 pints of 5% beer contains about 45 g alcohol	
Mori et al, 1992, Indian Health Service hospital, Pine Ridge Reservation, South Dakota, USA		Cases (46): All new, active, adult (18 years and above), cases registered between 1983–1989. Controls (46). Randomly selected, matched for age and residence, from health care register in Reservation, where all residents are included		Chart review: Alcohol abuse/alcoholism listed in medical record, or alcohol related admission within 10 years or outpatient visit within 5 years		Matched by age and residence. OR adjusted for sex, isoniazid profylaxis, and diabetes All study subjects from same Indian community.		Adjusted OR (AOR) for alcohol abuse vs. no alcohol abuse: 3.8 (1.15–12.3)		Prevalence in control group: 32%	
Buskin, et al, 1994, Seattle, King County Tuberculosis Clinic, Washington State, 1988–1990		Cases (151): Active TB cases, aged > 17 registered at TB clinic 1988–1990 Controls (545): Individuals seeking care at the clinic, but no TB diagnosed		Self reported frequency of drinking and amount consumed. Heavy drinkers defined as 3 or more drinks/day or more than 5 drinks on average on each drinking occasion.		OR adjusted for age and smoking. Sex, SES, BMI, and race were analysed, but did not influence result		Adjusted OR heavy drinking vs. non-drinkers 2.0 (95% CI: 1.1; 3.7)		1 US standard drink is 14 gram, thus 3 standard drinks is 42 gram. Prevalence of heavy drinking in control group: 12.5%	
Rosenman et al,1996, New Jersey, USA, 1985–1987		Cases (148): All active male, HIV-negative, cases over age of 35, born in USA, notified 1985–87 Controls (290): From Medicade finance administration files, matched for age and race		Self reported. "Heavy drinking" defined as > 22 alcohol equivalents/week		Only HIV- men in study, controls matched for age and race. Alcohol association not controlled for other variables in study, since alcohol was treated purely as confounder		Crude OR: 3.33 (1.99.5.59)		Prevalence "heavy drinkers" among controls: 14% 1 US standard drink is 14 gram, thus > 22 drinks per week = > 44 grams per day	
Schluger et al, 1999, Social services agencies and chest clinic, NY, USA. 1994–1997		Cases (20): Persons screened positive for active TB among 3,828 individuals seeking social services Controls (3,245): Those not screened positive for active TB		Self reported "moderate to heavy alcohol use". This was not defined further		None, but all subjects are social service clients		Crude OR 2.38 (0.88–6.58)		The authors did analyse, the study as a case control study. Considering that the subjects were all social service clients and alcohol problem was common in this group, it can be assumed that "moderate to heavy" correspond to at least 40 g per day and/or alcohol abuse Prevalence among controls: 43%	
Spletter, 2000, TB Control Clinic, Phoenix, Ariziona, USA, 1993–1999		Cases (43): active pulmonary TB, 25–64 years old, excluding refugees, HIV positive, and comorbidity such as gastrectomy, jejunuilial bypass, DM, silicosis, renal failure, immunosuppressive treatment, malignancies. Controls (258): Patients infected with *M.tuberculosis*, but active disease ruled out.		Medical record review: Heavy drinking defined as those with chart entries indicating alcohol abuse or alcohol history recorded as "heavy drinking"		See list of exclusion criteria. Controlled for age, sex, smoking, race, US born, high risk residence, illicit drug use.		Adjusted OR for heavy alcohol use vs. no heavy alcohol use: 6.1 (1.4; 26.2):		Entire study population are TB infected. OR reflect risk of progress to active disease. Prevalence of heavy alcohol consumption in controls: 2.3%	
Dong et al, 2001, 12 communes in Chengdu, China, 1996–97		Cases (174): All active TB cases recorded between March 1996 and March 1997 Controls (174): Random sample from community (population registry), matched for age, sex, and place of residence		Self reported use. Definition of alcohol use or amounts not reported.		Matched for age and sex and district. Smoking, crowding, darkness in dwelling, air-pollution and BMI are reported variables, but not reported what was actually controlled for in the logistic regression		Adjusted OR (alcohol vs no alcohol): 1.76 (0.90–3.42)		
Tocque et al 2001, Liverpool, UK, 1989–1996		Cases (112): All notified in the city Controls (198): From Liverpool general practitioner database, matched for sex, age and residential area		Self reported, high consumption defined as > 30 units per week (> 4.3/day), both at time of interview and 2 years prior to diagnosis		Matched for age, sex, and residence area Alcohol not included in multivariate analysis		Crude OR for drinkers vs. non-drinkers: 1.01 (0.67–1.70, at 2 years before diagnosis		One UK alcohol unit is 8 gram, thus 4.3 units/day = 34 gram	
Tekkel et al, 2002, Hospital, Tallinn, Estonia, 1999–2000		Cases (248): consecutive, incident pulmonary TB cases admitted to one hospital in Tallinn Controls (248): From population registry, matched for age, sex, and country of residence.		Self reported frequency of drinking during last year. Not defined in amounts of alcohol		Age, sex, and country of residence matched for. OR adjusted for smoking, drug abuse, nutrition, weight loss, contact with TB, place of birth, marital status, and education		Adjusted OR for people who consumed alcohol several times a week/day vs. rarely: 13.63 (4.63–40.10);		Prevalence of alcohol consumptions several times per week: 7.3%	
Crampin et al, 2004, Karonga district, Malawi, 1996–2001		Cases (598):All new TB cases, aged > 15, residing in district Controls (992): Random sample from community register, matched for age, sex and areas of residence.		Self reported as current (1/week or < 1/week), past, or never		Matched for age, sex, area of residence. Adjusted for SES, HIV, TB contacts, BCG		Adjusted OR for current 1/w vs. never: 0.9 (0.5–1.7)		Prevalence of drinking 1/week among controls: 11%	
Kim and Crittenden, 2005, County Prison, USA, 1992–1998		Cases (441): All inmates screened positive for active TB 1992–1998 Control (478): Sex matched, random sample from prison pop.		Alcohol abuse as recorded in prison health record		Sex, age, ethnicity, marital status, education, homelessness, IV drug use, HIV, length of stay in prison, type of crime.		Adjusted OR for alcohol abuse vs. no alcohol abuse: 1.59 (p < 0.01, no confidence interval reported)		Prevalence of alcohol abuse among controls: 40.2%	
Lienhardt et al 2005, Multicenter, Guinée, Guniea Bissau, and The Gambia, 1999–2001		Cases (687): Newly detected smear positive TB Controls: For each case: A (687): Age-matched household control, and: B (687): Residence area matched community control		Self reported as never/past/current		A large set of host related and environmental factors		Crude OR for current/past vs. never: 1.84 (1.28–2.66)		When controlling for age, sex, family history of TB, HIV and smoking, this association was no longer significant. However, no adjusted OR is reported in paper. Prevalence of current/past use among controls: 19%	
Selassie et al: 2005, South Carolina, USA, 1970–2002		Cases (437): All recurrent pulmonary TB cases, after at least 12 months from time of treatment completion between 1970 and 2001 Controls (442): Random sample of people who remained free of TB > 12 months after completion, matched for year of initial diagnosis		Medical records reviewed. "Alcoholism" as recorded in medical record		Age, sex, race, treatment duration, adherence, regimen, HIV/AIDS, other chronic condition, country of residence, initial sputum, reported side effects.		Adjusted OR for alcoholism vs. no alcoholism: 3.90 (2.49–6.12)		Entire study population are TB infected and previously successfully treated. OR reflect risk of recurrent TB. Prevalence of recorded alcoholism among controls: 12.4%	
Riekstina, et al 2005, Latvia, 1996		Cases (48): New pulmonary cases who had early (within 4 years) recurrence after successful treatment, adults only, excluding those with any resistance to first line drugs, and prisoners Control (96):successful treatment, no recurrence, matched for sex and bacteriological status		Alcohol problem according to medical records		Sex and bacteriological status matched for. Age, sex, unemployment, treatment facility, treatment interruption		Adjusted OR for alcohol problems vs. no alcohol problem: 16.63 (3.63–76.10)		Entire study population are TB infected. OR reflect risk of progress to active disease. Prevalence among controls (all TB patients): 23%	
Shetty et al, 2006, Medical college hospital, Bangalore, India, 2001–2003		Cases (189): all consecutive new active pulmonary TB Controls (189): age and sex matched relatives of non-TB patients in same hospital		Self reported as never, past (> 6 months ago), or current use. Amounts not reported.		Age and sex matched. Education, income, crowding, religion, marital status, BMI, cooking fuel, smoking, chronic illness.		Adjusted OR for current vs.- never use 2.37 (0.95–5.93)		Prevalence of current alcohol use in control group: 11.1%	
Coker et al, 2006, TB clinic, Samara town, Russia, 2003		Cases (334): Culture confirmed pulmonary TB Controls (334): Age and sex matched from population registry		Self reported "heavy drinking" at least once per month during last year, but "heavy drinking" not further defined		Age and sex matched. Adjusted for exposure (family contact and drinking unpasteurized milk)		Adjusted OR for heavy drinking at least once a month vs. no drinking: 2.43 (1.22–4.85)		Not clear if also smoking, illicit drug use, imprisonment, and household assets were controlled for. Alcohol not included in final multivariate analysis, reason not reported, alcohol listed as "not appropriate" in table.	
Kolappan et al, 2007, Prevalence survey 2001–2003, Rural district, Tamil Nadu, India		Cases (429): Bacteriologically positive cases, aged > = 15, detected during prevalence survey Controls (93,516): Those not diagnosed with TB in the prevalence survey, aged > = 15		Self reported, alcohol intake in ml. Alcoholism not defined.		Age, sex, smoking		Adjusted OR for alcoholism vs. no alcoholism: 1.5 (1.2–2.0)		Prevalence among controls: 11%	

OR = Odds Ratio, DM = Diabetes Mellitus, BMI = Body Mass Index, TST = Tuberculin Skin Test, SES = Socioeconomic Status

The studies were initially grouped in three categories with regards to exposure level. The low-exposure category (4 studies) included those studies that defined exposure as alcohol use above a cut-off point that was set at a level below 40 g (or 50 ml) alcohol per day. This is the upper cut-off point for low-risk (for chronic harm) alcohol consumption for men [46]. The high-exposure category (5 studies) included studies that defined exposure as alcohol consumption above a cut-off set at a level above 40 g per day. The third category included 6 studies that had ascertained a diagnosis of alcohol use disorder from medical records. None of the studies included details about any ICD classification used for the diagnosis. Therefore, this categorization is imprecise and does not allow for further subgroup analysis with regard to alcohol use disorders. One study[27] included data which allowed calculation of crude odds ratio for both alcohol consumption above 40 g/day, and for consumption between 10 and 40 g/day. The data were included in the low- and high-exposure categories respectively (labelled Brown I and Brown II respectively). Seven studies that did not report how exposure had been defined were excluded from this categorization.

Adjusted odds ratios for the odds of active TB disease among people with a particular level/type of alcohol exposure vs. no such exposure were extracted from the original papers. If no adjusted odds ratio (results reported from multivariate or stratified analyses) were reported, crude odds ratios were either extracted, or calculated from absolute numbers reported in the paper. Heterogeneity was assessed using Cochrane's Q statistic and the I^2^ statistic which estimates the percentage of the total variation across studies that is due to heterogeneity rather than chance [47]. Pooled effect sizes were calculated using both fixed and random effect models for each sub-category of studies.

The pooled effect size across the five high-exposure category studies did not differ significantly from the pooled effect size across the six studies that had ascertained a diagnosis of an alcohol use disorder. Therefore, these two categories were combined into one high-exposure/alcohol use disorder category (herewith termed "high-exposure category") for the further analysis. Those 11 studies were further grouped with regards to which constellation of confounders had been controlled for (age, sex, HIV, smoking, socioeconomic status (SES), and infection status), and according to types of TB studied (table 2). None of the studies reported disaggregated analysis by type of TB. Therefore, further subgroup analyses with regards to type of TB was not possible. In Table 2 we have reported the results of models with fixed and random effects but we will refer to the results of the random effects models in the following discussion. Although the random effects model gives slightly higher estimates of the effect sizes than does the fixed effects model it also gives wider confidence limits and the confidence limits for the latter are always contained within the confidence limits for the former.

**Table 2:** Pooled effect sizes for different sub-categories of studies.

**Study category**	**No of studies**	**Hetero-geneity test ** Cochrane's Q p-value (I^2^)	**Pooled, fixed effect assumption** **(95% confidence interval)**	**Pooled, random effect assumption** **(95% confidence interval)**
**Level of exposure**				
High exposure	11	< 0.01 (0.82)	2.90 (2.39–3.51)	3.50 (2.01–5.93)
Low exposure	4	0.46 (0.00)	1.08 (0.82–1.40)	1.08 (0.82–1.40)

**High-exposure studies**				
Controlled* for HIV status	7	0.03 (0.57)	2.93 (2.37–3.61)	3.26 (2.26–4.70)
Controlled* age, sex, SES, smoking	5	0.04 (0.61)	3.27 (2.38–4.50)	3.49 (2.06–5.90)
Controlled* HIV, age, sex, SES, smoking	4	0.07 (0.42)	3.92 (2.70–5.71)	4.08 (2.49–6.68)
Controlled* infection, age, sex, SES	4	0.23 (0.30)	4.11 (2.84–5.94)	4.21 (2.73–6.48)
Excluding three smallest studies	8	0.03 (0.59)	2.75 (2.19–3.46)	2.94 (1.89–4.59)
Excluding three smallest and Brown I and Kim	6	0.32 (0.15)	2.76 (2.34–3.81)	2.96 (2.28–3.85)
Pulmonary TB cases only**	2	0.49 (0.00)	3.67 (2.58–5.22)	3.67 (2.58–5.22)
All types of TB**	6	< 0.01 (0.83)	2.52 (1.98–3.19)	2.87 (1.47–5.58)

*Controlled for respective covariates, either by design (e.g. through inclusion/exclusion criteria) or in the analysis (stratification or multivariate analysis)

**Excluding three smallest studies

Funnel plots (log odds ratio plotted against the standard error of the log odds ratio for each study) were constructed to examine potential publication bias. Publication bias was suspected if relatively few studies with high standard errors and odds ratios close to one were identified[48]. Since publication bias was suggested by the funnel plots, we excluded the three studies with the highest standard errors for the analysis of pooled effect across studies in the high exposure category.

## Results

**Figure 1:**
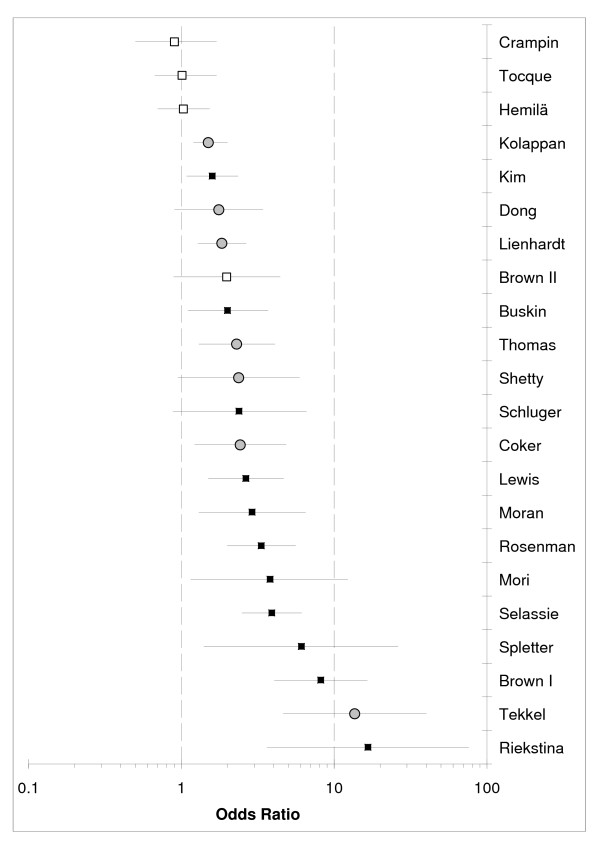
**Forest plot of all 21 studies.** Bars indicate 95% confidence interval. Filled squares represent point estimate for studies in the high exposure/alcoholism category, white squares represent studies in the low exposure category, and grey circles studies that did not report a well-defined exposure level.

Among the 21 reviewed studies, three studies were cohort studies and 18 were case control studies. Table 1 summarizes the characteristics of these studies. Figure 1 displays effect sizes in the 21 studies.

The pooled odds ratio across the 11 studies in the high-exposure category was 3.50 (95% CI: 2.01–5.93). The pooled odds ration across the four studies in the low-exposure category was (1.08, 95% CI: 0.82–1.40) (Table 2).

**Figure 2:**
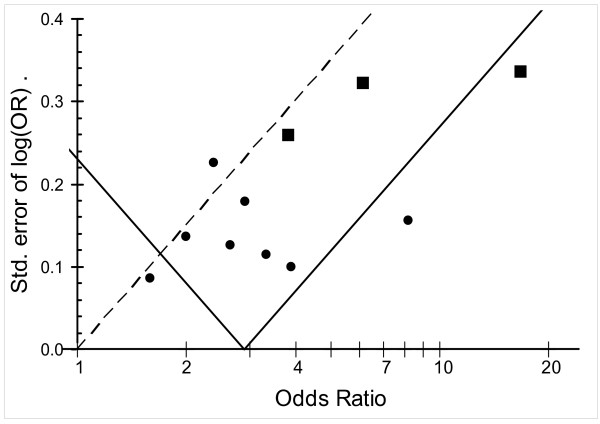
**Funnel plot of the odds-ratio against the precision of the estimates.** Points to the right of the dashed line are significant at the 5% level. The apex of the funnel gives the point estimate. Points outside the funnel differ from the point estimate at the 5% level and suggest heterogeneity in the estimates. If there is no bias in the selection of studies for publication, the points should be evenly scattered to the left and right. Squares represent the three studies with largest standard error that were excluded in the category "Excluding three smallest studies" in table 2 (Mori et al 1992, Spletter 2000, and Riekstina et al 2005). The two filled circles that are outside the funnel represent the two additional studies that were excluded in the category "Excluding three smallest and Brown I and Kim" in table 2

Funnel plots indicated that there was under-representation of small studies with weak or absent association, both for all studies combined, as well as for the studies in the high-exposure category (figure 2).

After exclusion of the three studies that had the highest standard error, because of suspected publication bias, the pooled effect sizes for studies in the high-exposure category was 2.94 (95% CI 1.89–4.59). There was significant heterogeneity across these studies. When further excluding the two studies with the highest and lowest effect sizes respectively (Brown I and Kim), there was no heterogeneity and the pooled effect size was 2.76 (95% CI 2.09–3.64).

Studies that included only pulmonary TB cases had higher pooled odds ratio than studies that included all types of TB. The difference was of borderline statistical significance (OR 4.16, 95% CI: 2.99–5.80 vs. 2.55, 95% CI: 2.02–3.23). After excluding the three smallest studies, the difference between the pooled odds ratio for these two categories decreased and was not statistically significant (3.67, 95% CI: 2.58–5.22, vs. 2.52, 95% CI: 1.98–3.19, table 2). Studies in the high-exposure category that had controlled for different sets of important confounders had similar or higher, but not significantly different, pooled effect size compared to all studies in this category combined (table 2).

## Discussion

This review suggest that low to moderate alcohol intake is not associated with increased risk of TB disease. However, there seem to be a substantial risk increase among people who drink more than 40 g alcohol per day, and/or have an alcohol use disorder. The pooled effect size across studies in the high-exposure category was 2.94 (95% CI 1.89–4.59) after excluding the three studies with largest standard errors in order to make a crude adjustment for the suspected publication bias. There was a tendency that studies that included only pulmonary TB cases reported higher odds ratios than studies that included all types of TB, but the difference was not statistically significant when small studies had been excluded to adjust for possible publication bias.

The original heterogeneity across all 21 studies decreased after subdividing studies into low and high-exposure level studies. However, there was remaining significant heterogeneity in the high-exposure category, which we could not explain through further subgroup analysis. Varying degree of misclassification of exposure across the studies may explain some of the heterogeneity. However, there was insufficient information in the reviewed studies to explore this further. Underestimation of level of alcohol intake may have biased the results in several studies. It is reasonable to assume that underestimation of alcohol intake by study subjects was either non-differential, or more pronounced among the cases in the case-control studies. In either case this would have led to an underestimation of the risk increase.

Bias caused by different approaches for the selection of controls in the case control studies may also have contributed to the heterogeneity. Several of the case control studies used hospital controls or controls recruited among other groups, such as prisoners and social service clients, that are likely to have higher alcohol intake levels than the general population. This may have biased, to various degrees across the studies, the odds ratios towards one.

Studies that had controlled for potential confounding effects (either by design or in the analysis) of important factors such as age, sex, HIV, some measure of socioeconomic status, and smoking, had similar or somewhat higher, but not significantly different pooled effect sizes. The degree to which important confounders were controlled for varied considerably across studies. There might have been residual confounding that could have biased the pooled estimate across the studies. Socioeconomic status is difficult to measure and fully control for. Furthermore, there are some risk factors for TB disease that have not been assessed in most of the reviewed studies. For example, malnutrition[19], diabetes[49] and indoor air pollution[50] respectively are associated with higher risk of TB disease. Mental health disorders may also be associated with higher risk of TB through impact on the immune system[21,22]. However, confounding effect of these factors would have to be of considerable magnitude to offset the relatively strong association found in this review. Moreover, it may not be correct to control for some factors, since they may be on the causal pathway. For example, alcohol use disorders can lead to social downward drift and it can cause or contribute to malnutrition. Other known risk factors for TB such as silicosis, malignancies and immunosuppressant treatment are probably too rare to have influenced the results significantly.

The pooled effect size across high-exposure studies that had controlled for infection status (OR 4.21, 95% CI: 2.73–6.48), suggest that one possible causal pathway through which alcohol operates as a risk factor for TB, is through increased risk of progression from infection to disease. It is somewhat surprising that this pooled effect size is larger than for the pooled effect size across all studies. However, the confidence interval is wide, and overlaps that of the pooled effect of the other studies. Furthermore, it is possible that part of the risk increase is due to increased risk of re-infection, since none of the studies was designed to distinguish re-infection from re-activation. Four studies in the high-exposure category were designed as cohort studies, or nested case control studies, in a way that allowed controlling for infection status. All four studies defined exposure as an alcohol use disorder noted in medical records in a way that allowed ascertaining the temporal sequence between exposure and outcome. All four studies reported adjusted risk ratios controlled for age, sex, some indicator of socioeconomic status. Three of these studies also controlled for smoking and three controlled for HIV. Two of the studies used recurrent TB as study outcome, and both controlled for type of treatment, treatment duration, and adherence.

Alcohol may assert a direct toxic effects on the immune system rendering the host more susceptible to TB disease. Animal studies suggest that cell mediated immunity and macrophage functions (which are essential for the host response to *M. tuberculosis *infection) are directly impaired by chronic and acute alcohol consumption[14,15]. One mechanisms may be through inhibited tumour necrosis factor (TNF) response[18]. Alcohol may also reduce the NO system response to mycobacterial infection, which may prevent the destruction of mycobacteria. Furthermore, at least in mice, alcohol can inhibit granuloma formation, IL-2 production, IFN-gamma production, and CD4^+ ^proliferation[17]. Alcohol use disorders may also cause impaired immunity indirectly through micro- and macronutrient deficiency, or through other alcohol-related disorders such as malignancies[20].

The association between alcohol use and TB could also be explained by specific social mixing patterns, which may increase the risk of exposure to people with infectious TB disease in settings such as bars, shelters for homeless, prisons, and social institutions. This is supported by a few molecular-epidemiological studies. A study in a high incidence areas of Western Cape Province, South Africa, 1993–1996, suggested that most of the TB transmission took place outside the households, and found that 58% the identified contacts outside the household took place while drinking in social groups[11]. Zolnir-Dovc[12] demonstrated an increased risk of belonging to a TB cluster among people with alcohol use disorders, indicating increased risk of recent transmission. Diel et al[13] investigated a TB outbreak in Hamburg, Germany during 1997–2002. They demonstrated that transmission between people who were socialising in a specific bar was an important factor behind the dissemination and perpetuation of the outbreak. The increased risk of TB transmission in prisons has been well established[51]. The prevalence of TB among people in social service institutions have been found to be very high [8-10].

The strength of the association is likely to vary between settings, due to varied social context of alcohol use and different mix of other risk factors that could modify the effect of alcohol use. The random effect model for pooling effect sizes may be appropriate in this context, since it assumes an underlying variation of the true effect size across different settings. However, this variation may not be random, and we cannot draw conclusions about which factors might modify the effects in a systematic way. Therefore, generalization to a specific setting, even based on the confidence limits of the random effect model, should be done with caution.

Nevertheless, the pooled effect size can be used to obtain an indicative estimate of population level importance of alcohol use as risk factors for TB disease. There is a huge variation in prevalence of drinking more than 40 gram alcohol per day (for men, and > 20 g per day for women) across the world, ranging from 0.1% in parts of the Eastern Mediterranean Region to 18.6% in parts of Eastern Europe [46]. The population attributable fraction can be calculated from these prevalence estimates and from the odds ratio obtained in the present study (2.9, 95% CI: 1.9–4.6): It ranges from close to zero in parts of the Eastern Mediterranean Region to more than 30% in parts of Europe.

Future research on the association between alcohol use and risk of TB should carefully assess both potential confounding effects and interaction between alcohol use and other TB risk factors. Possible difference in the risk of pulmonary vs. non-pulmonary TB should also be investigated. Furthermore, there is a need to better understand the possible causal pathways with regards to risk of infection and risk of break down from infection to disease.

## Conclusion

There is a three-fold risk increase of active TB associated with consumption of more than 40 g alcohol per day, and/or having an alcohol use disorder. This could be due to both increased risk of infection related to specific social mixing patterns associated with alcohol use, as well as influence on the immune system of alcohol itself and of alcohol related conditions. These findings have implications for TB control strategies globally, particularly in countries where a high proportion of TB can be attributed to alcohol use.

## Competing interests

The authors declare that they have no competing interests. Four of the authors (KL, BW, EJ and CD) are staff members of the World Health Organization. The authors alone are responsible for the views expressed in this publication and they do not necessarily represent the decisions or policies of the World Health Organization. No external funding was provided for this research.

## Authors' contributions

All authors contributed to the conceptualisation of the paper. KL and SS did the initial review, the selection of abstracts, and the identification of papers to be included in the final review. All authors contributed to the assessment of papers. BGW and KL did the statistical analysis. All authors reviewed the results of the analysis. KL drafted the manuscript, and all authors contributed to its completion. KL is the guarantor.

## Pre-publication history

The pre-publication history for this paper can be accessed here:


